# The potential of fungi in the bioremediation of pharmaceutically active compounds: a comprehensive review

**DOI:** 10.3389/fmicb.2023.1207792

**Published:** 2023-07-12

**Authors:** Ayodeji Amobonye, Christiana E. Aruwa, Sesan Aransiola, John Omame, Toyin D. Alabi, Japareng Lalung

**Affiliations:** ^1^School of Industrial Technology, Universiti Sains Malaysia, Penang, Malaysia; ^2^Department of Biotechnology and Food Science, Faculty of Applied Sciences, Durban University of Technology, Durban, South Africa; ^3^Bioresources Development Centre, National Biotechnology Development Agency, P.M.B. Onipanu, Ogbomosho, Nigeria; ^4^National Environmental Standards and Regulations Enforcement Agency, Lagos Field Office, Lagos, Nigeria; ^5^Department of Life Sciences, Baze University, Abuja, Nigeria; ^6^Centre for Global Sustainability Studies, Universiti Sains Malaysia, Penang, Malaysia

**Keywords:** bioremediation, degradation, environment, fungi, drug compounds, pharmaceutically active compounds, pollution, wastewater

## Abstract

The ability of fungal species to produce a wide range of enzymes and metabolites, which act synergistically, makes them valuable tools in bioremediation, especially in the removal of pharmaceutically active compounds (PhACs) from contaminated environments. PhACs are compounds that have been specifically designed to treat or alter animal physiological conditions and they include antibiotics, analgesics, hormones, and steroids. Their detrimental effects on all life forms have become a source of public outcry due their persistent nature and their uncontrolled discharge into various wastewater effluents, hospital effluents, and surface waters. Studies have however shown that fungi have the necessary metabolic machinery to degrade PhACs in complex environments, such as soil and water, in addition they can be utilized in bioreactor systems to remove PhACs. In this regard, this review highlights fungal species with immense potential in the biodegradation of PhACs, their enzymatic arsenal as well as the probable mechanism of biodegradation. The challenges encumbering the real-time application of this promising bioremediative approach are also highlighted, as well as the areas of improvement and future perspective. In all, this paper points researchers to the fact that fungal bioremediation is a promising strategy for addressing the growing issue of pharmaceutical contamination in the environment and can help to mitigate the negative impacts on ecosystems and human health.

## 1. Introduction

Pharmaceutical active compounds (PhACs) are a major class of emerging pollutants which comprise of small molecule pharmaceuticals (antibiotics, analgesics, diuretics, tranquilizers, psychiatric drugs, etc) as well as biologics (anti-toxins, blood products, hormones, interleukins, monoclonal antibodies, vaccines, etc.) (Rodrigues et al., 2023). They are key components of both human and veterinary medicine. However, PhACs are remarkably stable and not fully metabolized in both human and animal systems, thus, they eventually end up in the environment (González-González et al., 2022). The removal of the released PhACs from the environment, especially from wastewater, has been noted to be a very challenging task by various authors (Castiglioni et al., 2006; Papagiannaki et al., 2022; Rodrigues et al., 2023). This is borne out of the fact that PhACs have a low octanol/water partition coefficient, which indicates their high solubility and polarity. In addition, they are highly mobile and are quite resistant to biodegradation under ambient conditions (Christensen et al., 2022). Consequently, these pollutants persist perpetually in various water bodies across the environment. In this regard, concentrations of PhACs ranging from ng/L–μg/L have been recorded in effluents from sewage treatment plants, sediments, surface water, ground water, and occasionally in drinking water supplies (Papagiannaki et al., 2022). For instance, in a recent study by He et al. (2022), different levels of antibiotics including ciprofloxacin, erythromycin, ciprofloxacin, roxithromycin, sulfadiazine, sulfamethoxazole, tetracycline, and oxytetracycline, as well as analgesics, ibuprofen, naproxen were recorded in drinking water sources in a city in China.

The risk posed by the persistence of PhACs in the environment cannot be overemphasized. These risks range from development of antibiotic resistance, damage to the aquatic life, hormonal disruption, and bioaccumulation to low quality drinking water (Dos Santos et al., 2021; He et al., 2022; Papagiannaki et al., 2022). Unfortunately, the efficiency of PhACs removal through conventional wastewater treatment techniques such as adsorption, membrane filtration, ozonation, photolysis, photocatalysis has been noted to be limited due to one or many reasons (Cai et al., 2018). For instance, although membrane filtration via nanofiltration and reverse osmosis have been identified as effective in removing low molecular weight PhACs, the large-scale deployment of this technique is critically limited by membrane fouling which results in high operational cost (Cornelissen et al., 2021). Similarly, ozonation, which has high potential as a PhACs secondary treatment method is noted to be highly energy demanding and may also lead to the generation of oxidation products/intermediates with higher toxicity as recently established by Quaresma et al. (2021). In this regard, the search for environmentally friendly and effective treatment processes to remediate PhACs in the environment is critical (Narayanan et al., 2022).

Hence, various research efforts have been placed on remediating PhACs and other emerging contaminants from the environment using biological methods based on different prokaryotic and eukaryotic systems that have been identified with high with recoverability and reusability potential (Bilal et al., 2019). Generally, these organisms have rapid multiplication rates, short generational time, and flexible genetic machinery, which in all, enable them to evolve their metabolic capacity to allow the incorporation of new compounds into their metabolic pathways (Amobonye et al., 2021). Furthermore, their ability to adapt to the metabolism of novel anthropogenic compounds including PhACs is believed to be based on the natural selection of organisms which have developed the necessary degradative enzymes with less specific substrate-specificities and probably novel metabolic pathways (Amobonye et al., 2021). In this regard, organisms from all classes ranging from bacteria – *Chryseobacterium taeanense, Rhizobium daejeonense, Pseudomonas moorei, Nitrosomonas europaea*, etc. (Xu et al., 2016; Nguyen et al., 2019) to microalgae – *Chlorella sorokiniana, Chlorella vulgaris, Chlamydomonas Mexicana, Microcystis aeruginosa,* etc. (Xiong et al., 2018; Zhou et al., 2022) and fungi- *Ganoderma lucidum, Phanerochaete chrysosporium, Trametes versicolor* (Silva et al., 2019; Del Álamo et al., 2022) have been highlighted to facilitate the bioremediation of PhACs as individuals or in consortia.

Fungal bioremediation has since been identified as an effective biotechnology tool in the removal of various pollutants from the environments, this is in addition to the application of fungal organisms in the food, pharmaceutical, textile, paper and construction industries, to mention a few. Various studies have shown that various fungi as well as their enzymes, are important agents in the removal of pharmaceutical compounds and other persistent pollutants in various aquatic systems (Ferrando-Climent et al., 2015; Narayanan et al., 2022). Their effectiveness in this regard has been ascribed to their inherent ability to secrete a wide range of enzymes, including laccases, peroxidases, cytochrome P450 mixed function oxidases which transform the PhACs via reduction, oxidation, hydroxylation, dehalogenation, dehydrogenation, deamination, formylation, etc. (Ferrando-Climent et al., 2015; Narayanan et al., 2022). Recently, the abilities of *Fomes fomentarius, Hypholoma fasciculare, Phyllotopsis nidulans, Pleurotus ostreatus,* and *Trametes versicolor* to remove the cytostatic drugs, bleomycin and vincristine were described (Jureczko and Przystaś, 2021). Similarly, the biodegradation of ofloxacin along with some other medical chemicals in hospital wastewaters by *T. versicolor* was also demonstrated (Gros et al., 2014), while laccase enzyme from the same fungus was shown to degrade carbamazepine, diclofenac, sulfamethoxazole and trimethoprim (Alharbi et al., 2019).

Therefore, this paper highlights the recent findings on the roles of fungi as well as their enzymes in the bioremediation of PhACs. Emphasis has also been placed on the elucidating PhACs as emerging contaminants, and the effectiveness of specific fungal species in the removal of PhACs from the environment. This paper also describes the fungal enzyme machineries involved in PhACs bioremediation together with a probable mechanism for fungal bioremediation of PhACs. Different fungal-based bioreactors for PhACs degradation were also discussed. In addition, the future areas of development regarding this technology were also highlighted. This paper is expected to be an important reference for researchers in charting a new course for the fungal war against PhACs bioaccumulation.

## 2. Pharmaceutical active compounds as emerging micropollutants

**Figure 1:**
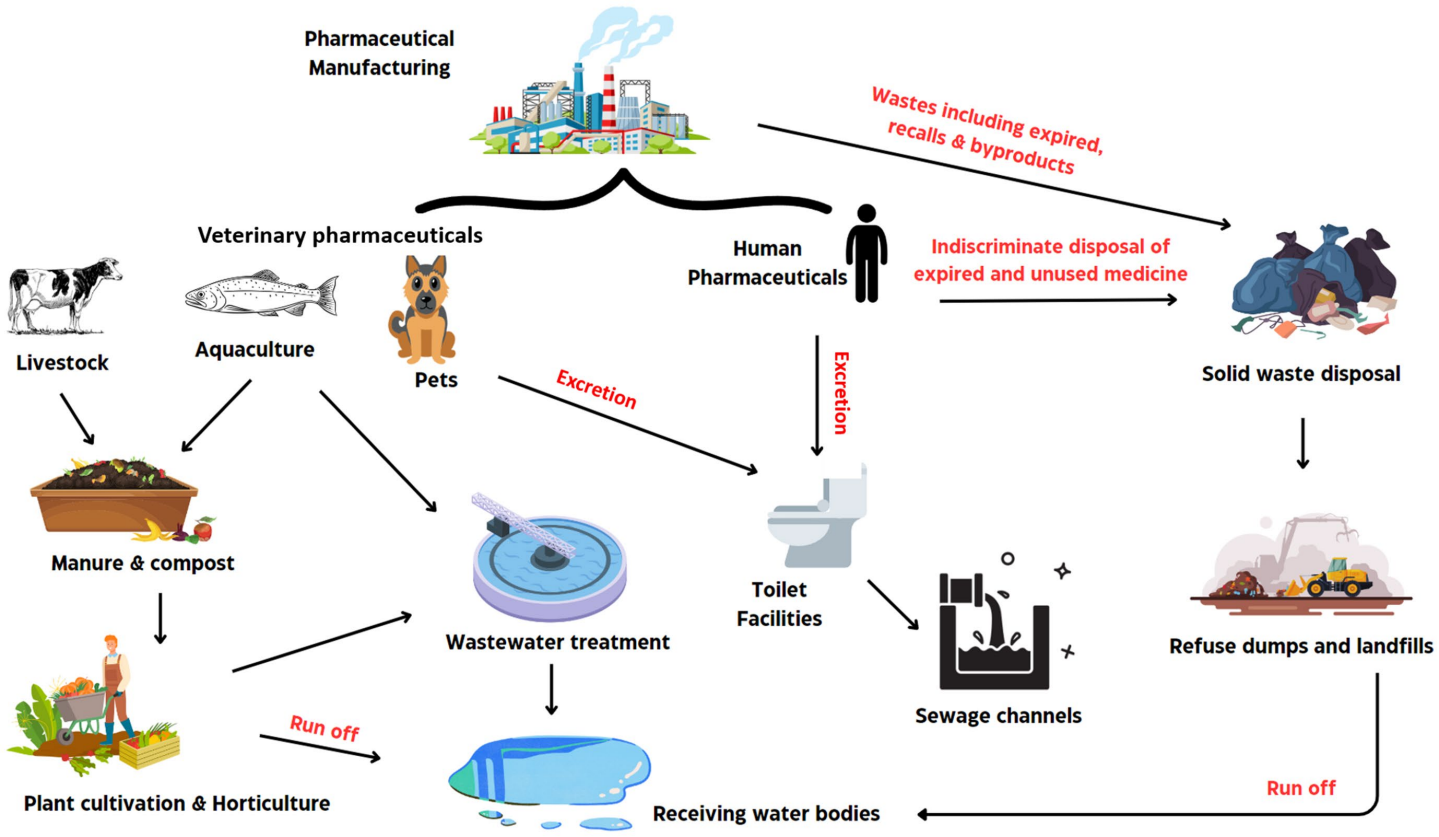
Sources and pathway of pharmaceutically active compounds in the environment.

The increased demand in the use of pharmaceutical active products is positively correlated with their rate of disposal into the environment, consequently these products constitute a substantial proportion of emerging micropollutants, and their effect on human health and the general environment cannot be overestimated (Silva et al., 2015; Akerman-Sanchez and Rojas-Jimenez, 2021). Hence, the presence PhACs across different ecosystems is raising public concern, due to their abundance, diversity, and their persistence in the environment. It was noted that more than 70 PhACs were present in wastewater treatment plant effluents at micrograms concentrations, posing an extended risk to human health and aquatic life (Rosal et al., 2010; Ramírez-Morales et al., 2020). PhACs are biologically active formulations that are broadly used as therapeutic agents in humans and livestock (Podolsky, 2018; Akerman-Sanchez and Rojas-Jimenez, 2021). They are specifically designed to treat or alter animal physiological conditions and they include antibiotics, analgesics, anti-inflammatories, antihypertensives, hormones, steroids, antipyretics, and stimulants. An overview of selected PhACs found in the environment as pollutants is given in Table 1. The excessive use of antibiotics and other PhACs, especially in livestock breeding and in aquaculture, has resulted into a significant increase in the levels of PhACs been released to the environment (Van Boeckel et al., 2015). According to Castiglioni et al. (2006), more than 50% of administered PhACs may be unmetabolized in the user and these are excreted in their original forms or as active metabolites into the environment via urine or/and stools. Although the major route of PhACs into the aquatic environment has been highlighted to be via human and animal excretion, ther other routes, though less important, have been identified to include disposal of unwanted or expired drugs, landfill leachates, agricultural activities, manufacturing processes, concentrated animal feeding operations as well as and urban run-off (Papagiannaki et al., 2022). A schematic representation of the different routes of entry of PhACs into the environment is presented in Figure 1.

**Table 1:** Properties of some pharmaceutical active compounds.

Pharmaceutical active compound	Average half-life h	Solubility	DrugBank accession number	Reference
*Analgesics*				
Aspirin	4.5	10 mg/mL	DB00945	Izadi et al. (2020)
Acetaminophen	2.5	4.15 mg/mL	DB00316	Izadi et al. (2020)
Diclofenac	2.0	2.37 mg/L	DB00586	Mlunguza et al. (2019)
Fenoprofen	3.0 h	NA	DB00573	Tyumina et al. (2020)
Ibuprofen	1.6 h	21 mg/L	DB01050	Mlunguza et al. (2019)
Indomethacin	4.5	0.0024 mg/mL	DB00328	Tyumina et al. (2020)
Ketoprofen	1.2	51 mg/L	DB01009	Mlunguza et al. (2019)
Naproxen	14.5	15.9 mg/L	DB00788	Tyumina et al. (2020)
*Antibacterial*				
Ampicillin	NA	1.1 × 10^−4^ mg/L	DB00415	Danner et al. (2019)
Amoxicillin	1.1	0.958 mg/mL	DB01060	Kovalakova et al. (2020)
Azithromycin	68	0.514 mg/mL	DB00207	Akhtar and Mannan (2020)
Cefalexin	0.83	10 mg/mL	DB00567	Danner et al. (2019)
Chloramphenicol	2.5	2,500 mg/L	DB00446	Gothwal and Shashidhar (2015)
Clarithromycin	~ 4	0.217 mg/mL	DB01211	Kovalakova et al. (2020)
Clindamycin	3	3.1 mg/mL	DB01190	Gothwal and Shashidhar (2015)
Danofloxacin	NA	0.738 mg/mL	DB11393	Gothwal and Shashidhar (2015)
Doxycycline	4	50 mg/mL	DB00254	Gothwal and Shashidhar (2015)
Gentamicin	1.25	12.6 mg/mL	DB00798	Gothwal and Shashidhar (2015)
Erythromytin	3.5	2 mg/mL	DB00199	Kovalakova et al. (2020)
Levofloxacin	7.0	1.44 mg/mL	DB01137	Zhang et al. (2023)
Metronidazole	8.6	5.92 mg/mL	DB00916	Danner et al. (2019)
Norfloxacin	3.5	1.78 × 10^−5^ mg/L	DB01059	Kovalakova et al. (2020)
Ofloxacin	9.0	28.3 mg/mL	DB01165	Zhang et al. (2023)
Oleandomycin	NA	0.41 mg/mL	DB11442	An et al. (2023)
Oxytetracycline	NA	313 mg/L	DB00595	Kovalakova et al. (2020)
Rifamycin	3.0	0.0147 mg/mL	DB11753	An et al. (2023)
Roxithromycin	12.0	0.0189 mg/L	DB00778	Gothwal and Shashidhar (2015)
Sarafloxacin	NA	0.105 mg/mL	DB11491	Gothwal and Shashidhar (2015)
Sulfamethoxazole	10.0 h	610 mg/L	DB01015	Gothwal and Shashidhar (2015)
Sulfapyridine	10.0 h	268 mg/L	DB00891	An et al. (2023)
Tetracycline	9.0 h	231 mg/L	DB00759	Kovalakova et al. (2020)
Trimethoprim	9.0	400 mg/L	DB00440	Kovalakova et al. (2020)
Vancomycin	6.0	0.225 mg/mL	DB00512	Gothwal and Shashidhar (2015)
*Antifungal*				
Amphotericin B	>24 h	750 mg/L	DB00681	Chang and Gupta (2022)
Bifonazole	1.5 h	0.00245 mg/mL	DB04794	Akhtar and Mannan (2020)
Caspofungin	10.0	0.367 mg/mL	DB00520	Monapathi et al. (2021)
Clotrimazole	NA	0.49 mg/L	DB00257	Akhtar and Mannan (2020)
Flucytosine	3.6	1.5 × 10^−4^ mg/L	DB01099	Assress et al. (2021)
Ketoconazole	8.0	9.31 × 10^−3^ mg/mL	DB01026	Assress et al. (2021)
Miconazole	24	7.63 × 10^−4^ mg/mL	DB01110	Assress et al. (2021)
Natamycin	NA	4,100 mg/L	DB00826	Monapathi et al. (2021)
Nystatin	NA	360 mg/L	DB00646	Monapathi et al. (2021)
*Hormones*				
Cortisol	2.15	320 mg/L	DB00741	Ojoghoro et al. (2021)
Dexamethasone	4.0	89 mg/L	DB01234	Ojoghoro et al. (2021)
Diethylstilbestrol	NA	12 mg/L	DB00255	Torres et al. (2021)
Estradiol	6.0	3.6 mg/L	DB00783	Torres et al. (2021)
Estrone	19.0	760 mg/L	DB00655	Morais et al. (2019)
Estriol	NA	0.119 mg/mL	DB04573	Morais et al. (2019)
Ethinyl estradiol	9.2	11.3 mg/L	DB00977	Morais et al. (2019)
Levonorgestrel	>24.0	2.05 mg/L	DB00367	Lasich and Adeleke (2023)
Methyltestosterone	7.0	33.9 mg/L	DB06710	
Prednisone	2.5 h	NA	DB00635	Ojoghoro et al. (2021)
Progesterone	NA	5.46 × 10^−3^ mg/mL	DB00396	Lasich and Adeleke (2023)
*Psychiatric*				
Carbamazepine	14.0	0.152 mg/mL	DB00564	Akhtar and Mannan (2020)
Diazepam	72.0	50 mg/L	DB00829	Adeola et al. (2022)
Chlorpromazine	30.0	2.55 mg/L	DB00477	Escudero et al. (2021)
Risperidone	11.5	2.33 mg/mL	DB00734	Escudero et al. (2021)
Haloperidol	25.3	14 mg/L	DB00502	Cerveny et al. (2021)

**Figure 2:**
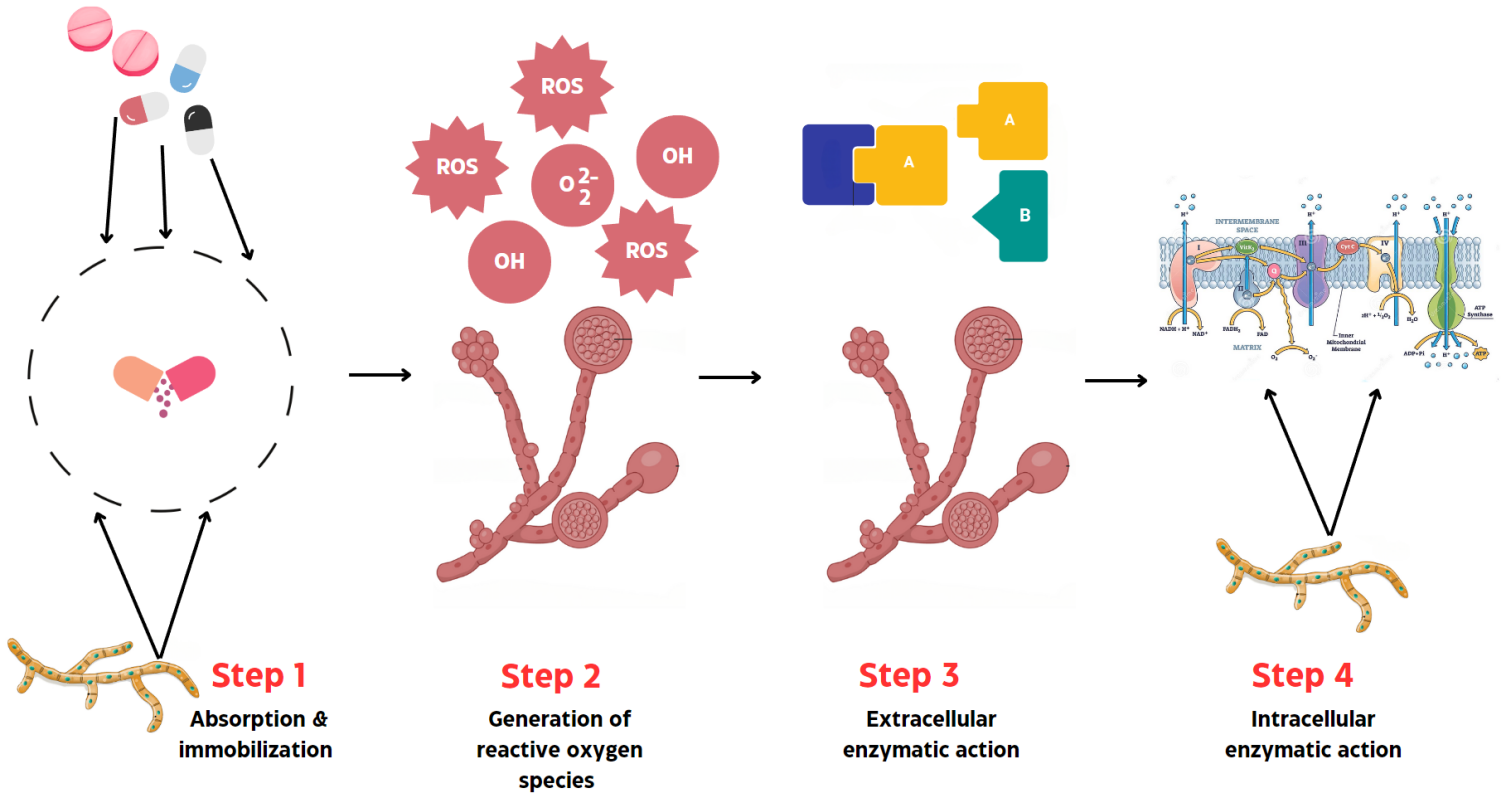
Mechanism of fungal bioremediation of Pharmaceutical Active Compounds.

It was noted that a significant amount of PhACs evade removal by conventional wastewater treatment plants (WWTPs) because these plants are basically designed for the removal of carbon and nutrients, in addition, the chemical nature of PhACs makes them recalcitrant to biological action (Vergili et al., 2019). Ramírez-Morales et al. (2020) was able to demonstrate high levels of PhACs in WWTP effluents which includes analgesics, antidiabetics, anti-inflammatory agents and psychiatric agents in concentrations ranging from 0.001 to 57 μg/L. In a different study, ofloxacin, erythromycin, ciprofloxacin, and roxithromycin, all antibiotics, were also detected in WWTP in concentrations up to 6.7 μg/L (Verlicchi et al., 2012). Consequently, the inability of conventional WWTP to completely remove PhACs has resulted into the onward transportation and emergence of these active contaminants in surface waters and in ground waters (Mahmood et al., 2019). Accumulation in the environment occurs to a point where PhACs are being detected in drinking water sources (Yang et al., 2017). This anomaly has been recorded in the most developed countries of the world, which are believed to be equipped with the latest technology in wastewater treatment, as well as in the less developed countries in the global south (Bexfield et al., 2019; Kondor et al., 2021). For instance, more than 120 PhACs, including carbamazepine, sulfamethoxazole, hydrocortisone, and meprobamate were recorded during the assessment of ~1,000 principal aquifers across the United States (Bexfield et al., 2019). Various PhACs including ciprofloxacin, sulfamethoxazole and triclosan (antibiotics), dexamethasone and diclofenac (anti-inflammatories), diazinon (antiparasitic drugs), primidone (antiepileptic), propranolol (beta-blockers), caffeine (psychoactive stimulants) were also detected in the range of < 0.03 to 21.39 ng/L in selected drinking water samples from Malaysia (Wee et al., 2020). Similarly in Nigeria, amoxicillin was detected in surface water, ground water and drinking water, at median concentrations of 1,614, 238, 358 ng/L, respectively, (Ebele et al., 2020). Varying concentrations of other PhACs including acetaminophen, caffeine, codeine, diclofenac glyburide, ibuprofen, naproxen and nicotine were also reported (Ebele et al., 2020). Thus, to further safeguard human health and the general environment, it has become imperative to consider the quantification of active pharmaceutical ingredients as critical components of water quality monitoring indicators (Figure 2).

Generally, the chemical structural diversity of pharmacological actives is extensive, reflecting the complexity of the human body and the wide range of biological targets with which medications might interact. PhACs have diverse chemical structures that range from aliphatic structures to heterocyclic and to aromatic. A majority of these pharmaceuticals have however been noted to be composed of heterocyclic and aromatic rings with fewer linear structures (Olicón-Hernández et al., 2017). In this regard, the complex structural components of PhACs affect their solubility and confers low-solubilization on them which in turns leads to low bioavailability. In addition to low bioavailability, most PhACs have antimicrobial properties which impede their biotransformation especially by using bacteria. Furthermore, the wide range in chemical structure of PhACs, make it difficult to elucidate general pathways for their microbial degradation (Olicón-Hernández et al., 2017). Thus, the PhACs usually encountered in the environment can be structurally classified into the main classes; aliphatic, aromatic, glycosides, heterocyclic, peptides (proteins), lipids (steroids), and nucleic acids Table 2.

**Table 2:** Fungal species with potential in Pharmaceutical Active Compound biodegradation.

S/N	Names	Source	Classification	PAM metabolized	Reference
1	*Aspergillus luchuensis*	Habitant is mainly in decay woods and close to fermentation site	Eurotiomycetes	Diclofenac	Dalecka et al. (2020)
2	*Aspergillus niger*	Soil, water, vegetation and decomposing matter	Eurotiomycetes	Diclofenac	Kasonga et al. (2021)
3	*Gymnopilus luteofolius*	Decay hardwood and conifers	Basidiomycetes	Lopromide, carbamazepine, diclofenac	Vasiliadou et al. (2016)
				Trinitrotoluene TNT	Anasonye et al. (2015)
4	*Irpex lacteus*	Dwells mainly in Angiosperm branches especially rotten parts of the wood	Agaricomycetes	Diclofenac, ibuprofen	Marco-Urrea et al. (2009)
5	*Mucor circinelloides*	Soil, dung and roots of some vegetables	Zygomycetes	Diclofenac, ibuprofen	Kasonga et al. (2019)
6	*Penicillium oxalicum*	Soil, decay vegetables, compost, dried food stuff.	Eurotiomyctes	Diclofenac	Kasonga et al. (2019)
7	*Rhizopus microspores*	Soil, Plant debris and food stuff	Zygomycetes	Carbamazepine, diclofenac, ibuprofen	Kasonga et al. (2023)
8	*Stropharia rugosoannulata*	Wood-chips beds and mulch mainly in garden areas		Carbamazepine, lopromide	Anasonye et al. (2015)
9	*Trametes versicolor*	Hardwoods such as beech and oak majorly as saprophytes	Agaricomycetes	Carbamazepine	Tormo-Budowski et al. (2021)
				Trimethoprin	Alharbi et al. (2019)
				Sulfamethoxazole	Stenholm et al. (2019)
10	*Trametes polyzona*	Decayed wood and soil	Agaricomyctes	Diclofenac	Kasonga et al. (2023)
11	*Trichoderma longibrachiatum*	Soil	Sordariomycetes	Diclofenac	Kasonga et al. (2023)

While the aromatic compounds such as aspirin, ibuprofen, and naproxen, are made up of aromatic rings, such as benzene, the heterocyclic compounds which includes caffeine, morphine, and penicillin are characterized by the presence of non-carbon atoms (nitrogen, oxygen, or sulphur) in their ring structure. Aliphatic pharmaceuticals such as acetaminophen, aspirin and ibuprofen, are based on straight chain structures and they contain neither aromatic nor heterocyclic rings (Carey et al., 2006). The peptides and proteins are large polymers of amino acids, and they include insulin and many growth hormones. Similarly, nucleic acids are also polymers made up of nucleotides, the building blocks of DNA and RNA and they include antiviral medicines and chemotherapeutic treatments (Pradeep et al., 2023). Glycoside pharmaceuticals are drug substances in which a sugar molecule is linked to a non-sugar molecule such as digitalis and heparin. On the other hand, lipids are characterized by their significant insolubility in water molecules that are crucial structural components of cell membranes, they include the steroids, cholesterol-lowering medications, and anti-inflammatory medications (Galán et al., 2019; Kumavath et al., 2021).

PhACs have been associated with bioaccumulation and biomagnification, endocrine disruption, carcinogenicity, nervous system degradation, dermal pathologies, and anti-biotic resistance (Olicón-Hernández et al., 2017). However, the effects of the re-introduction of these compounds into the human system have not been well delineated mainly because of the micro-level concentration of PhACs in the environment. On the other hand, many studies have described their detrimental effects on other life forms, especially on aquatic organisms and it is believed that findings from these studies could be used as basis to investigate human health effects upon environmental exposures (da Costa Araújo et al., 2019; Booth et al., 2020). PhACs with the potential to disrupt the endocrine system like synthetic oestrogen (excreted in urine and/or faeces as conjugates or unchanged parent compound) have been the subject of various scientific enquiry as they are excreted in urine and/or faeces either as a conjugate or unchanged as the parent compound. Subsequent to their metabolism in the environment, these estrogenic compounds become free biologically active PhACs in concentration which are believed to be sufficient to elicit estrogenic in animals (Arcand-Hoy et al., 1998). Various antibiotics have also been demonstrated to elicit various detrimental effects on organism in the aquatic environment which include growth inhibition, mutagenicity, oxidative stress, reproductive abnormalities, neurotoxicity and behavioural changes, to mention a few (Booth et al., 2020). For instance, exposure of gilthead seabream to erythromycin at between 0.0002 and 200 μg/L resulted into increased gill histopathological index while chronic exposure to oxytetracycline at a concentration of 0.0004–400 μg/L escalated its pathological index (Booth et al., 2020). Similarly, 30-day exposure of tadpoles to the anti-cancer pharmaceuticals, cyclophosphamide and 5-fluorouracil, at environmental concentrations of between 0.2 and 123 μg/L resulted in impaired visual acuity, mutagenicity and the development of melanocytes in gastrointestinal tract of tadpoles (Da Costa Araújo et al., 2019). To further elucidate the environmental occurrence, fate, and risks of PhACs different models such as the Fugacity-based multimedia modeling, FATEMOD-Q, iSTREEM, LF2000-WQX are now being used (Booth et al., 2020).

## 3. Fungal species in PhACs bioremediation

The fungi kingdom is a ubiquitous group well known for their phylogenetic diversity, their chemoheterotrophic nature, and their symbiotic interactions. They are also notable for their versatility in nutrients cycling as well as their decomposition of organic matter in nature (Hurdeal et al., 2021). These properties give fungal species a lot of edge over algae, actinomycetes and bacteria in bioremediative applications. As such, fungi’s biochemical and morphological attributes, especially the filamentous fungi, are being harnessed in the degradation of complex and emerging environmental pollutants like PhACs (Ferreira et al., 2020). Among the filamentous fungi, three groups have been majorly associated with pollutants decomposition. These include members of the ascomycetes (sac fungi), basidiomycetes (club fungi), and zygomycetes (conjugated fungi) (Ferreira et al., 2020). Studies have further shown that the group most widely associated with bioremediation are the white rot fungi (majorly basidiomycetes). The white rot fungi can break down PhACs with the help of their relatively advanced enzymatic systems which includes the lignin modifying enzymes (LMEs; Rodríguez-Rodríguez et al., 2013). Basically, the utilization of fungi in bioremediation, which is referred to as mycoremediation, is possible due to the unique ability of fungi to metabolize various inorganic and organic xenobiotics (use them as carbon and energy source), with the subsequent release of harmless metabolites or their complete assimilation.

### 3.1. Ascomycetes

Fungi within the Ascomycetes group are highly adaptive and are able to carry out metal ions chelation, a useful pathway in xenobiotic detoxification (Tigini et al., 2014). They are also capable of resisting unfavourable conditions and they exhibit fast growth even at near alkaline pH (Harms et al., 2011). Ascomycetes within the *Fusarium*, *Trametes* genera were shown to be the key contributors to PhACs degradation in a bioreactor system which ran effectively for 7 days (Badia-Fabregat et al., 2017). Their degradative ability is believed to be significantly mediated by the intracellular cytochrome oxidases, as well as by the expression of unspecific peroxygenases which may have an essential role in extracellular hydroxylation (Badia-Fabregat et al., 2017). Many of the species belonging to this phylum can also express the key lignin degrading enzymes. *Pestalotiopsis* sp. (IMI353656) was revealed to be involved in PhACs hydroxylation (Gonda et al., 2016), much like *Epicoccum nigrum* (IMI3542) which caused near total bioconversion of diclofenac to the 4-hydroxydiclofenac metabolite (Webster et al., 1998). The entomopathogenic fungus, *Beauveria bassiana*, has also been demonstrated to bio-transform cinoxacin via decarboxylation to hydroxy-methyl with dioxolo ring cleavage (Parshikov et al., 2002).

### 3.2. Basidiomycetes

Fungi within the Basidiomycete phylum possess oxidative enzyme systems which have been shown to be highly efficient in breakdown of pollutants (Naghdi et al., 2018). Their well-developed and non-specific lignin solubilizing enzymes enable their widened application for the degradation of PhACs and other emerging contaminants (Roccuzzo et al., 2021). As part of their oxidative enzyme system, laccases and peroxidases catalyse the non-specific oxidation of phenol-based aromatics (Aruwa et al., 2021, 2022), this is quite notable as many PhACs are composed of one or more phenols rings, which may or may not be fused (Ijoma and Tekere, 2017). Some Basidiomycetes that have been shown to possess the ability to bioremediate PhACs include *Bjerkandera adjusta*, *Ceriporiopsis subvermispora*, *Ganoderma lucidum*, *P. chrysosporium*, *Irpex lacteus*, *Trametes versicolor*, *Trametes hirsuta* and *Pleurotus ostreatus* (Mir-Tutusaus et al., 2018; Saibi et al., 2022). Furthermore, dye degrading fungi like *Phlebia tremellosa*, *Inonotus hispidus*, *Hirschioporus larincinus*, *Coriolus versicolor* are believed to play active roles in PhACs breakdown due to their LMEs (Deshmukh et al., 2016). *T. versicolor*, a model fungus in bioremediation, expresses highly versatile extracellular LMEs and intracellular cytochrome P450 enzymes (Marco-Urrea et al., 2010), hence its wide use in degradation of PhACs such as ibuprofen (Marco-Urrea et al., 2009), naxopren and carbamazepine (Rodríguez-Rodríguez et al., 2010). Members of this group have also been shown to act synergistically. For instance, fluoxetine and citalopram anti-depressants in wastewater effluents were efficiently degraded using a consortia of *P. chrysosporium* and *B. adusta* (Rodarte-Morales et al., 2011). Similarly, ionic/polar nitrogen-containing PhACs (diclofenac, indomethacin, naproxen, etc.) degraded by whole *T. versicolor* culture, its laccase and other intracellular enzymes’ machinery (Tran et al., 2010).

### 3.3. Zygomycetes

The Zygomycetes phylum is a diversified fungal group known for aplanospores (asexual phase) and zygospores formation (sexual phase). *Cunninghamella elegans* is more commonly referred to as a model Zygomycetes when assessing their xenobiotics degradative ability (Kandhasamy et al., 2022). This notable potential is believed to be linked to their capability of generating stereo- and regio-selective transformation. For example, *C. elegans* initially transformed PhACs through oxidative, reductive, and hydrolytic pathways to produce sulfoxidated and hydroxylated compounds, which may further be biotransformed to conjugated products (Kandhasamy et al., 2022). *C. elegans* also has been specifically shown to degrade a fibrate, gemfibrozil, a lipid regulating medication (Russell, 2004; Kandhasamy et al., 2022). Transformation of fluoroquinolones and carbamazepine by other Zygomycetes such as *Mucor rammanianus* and *Umbelopsis ramanniana* have also been reported (Kang et al., 2008). In a related study, a member of the Zygomycota phylum, *Mucor hiemalis* was found to degrade significant levels of acetaminophen under optimized conditions and could also be coupled with other bioremediation systems (Esterhuizen-Londt et al., 2016). Although the Zygomycetes have shown potential for PhACs bioremediation, however, little has been done about scaling-up these bioprocesses for real-time applications unlike the Basidiomycetes (Olicón-Hernández et al., 2017).

## 4. Fungal enzymes in PhACs bioremediation

Fungi, being one of the most important decomposers in nature, they are rich in different enzymes. Which catalyse the bioconversion of a variety of complex substrates. It was previously estimated that ~50% of the industrial enzymes currently in use were produced by fungi and they have found important applications in diverse industrial processes and products such as food, animal feed, pharmaceutical, textile, detergents, pulp and paper as well as bioremediation (Kango et al., 2019). Enzymes of fungal origin have been described with remarkable potential in the degradation of PhACs present in various waste streams under various conditions. As such, they thus offer a cost-effective and environmentally sustainable alternative to conventional treatment methods (Vaksmaa et al., 2023). This remarkable ability has been ascribed to their robustness, which allows them to degrade complex chemical structures into simpler and less toxic compounds that can be further metabolized by other microorganisms (Rathore et al., 2022). Furthermore, the biodegradation of non-polar and poorly soluble PhACs and other xenobiotics in organic solvents has been shown to be facilitated by these fungal enzymes (Espinosa-Ortiz et al., 2022). These enzymes modify and detoxify these pharmaceuticals via reduction, oxidation, hydroxylation, dehalogenation, dehydrogenation, deamination, formylation, etc. (Ferrando-Climent et al., 2015; Narayanan et al., 2022). In this regard a wide variety of fungal enzymes have been described for their roles in PhACs treatment, including laccases, peroxidases, cytochrome P450 mixed function oxidases, lipases, and esterases. However, the most studied fungal enzymes in PhACs biodegradation, *viz.*, laccases, peroxidases and cytochrome P450 mixed function oxidases will be discussed in detail in this section.

### 4.1. Laccases

Laccases (EC 1.10.3.2) are part of a superfamily of enzymes known as the multicopper enzymes; they were first described by Yoshida (1883), making them one of the earliest enzymes to be described. Most fungi have been shown to produce laccase, with laccases from *Agaricus bisporus*, *P. ostreatus*, *T. versicolor*, *P. chrysosporium*, and *Coprinus cinereus* being more prominent (Viswanath et al., 2014). Naturally laccases perform critical roles in lignin synthesis and in the degradation of plant cell walls as well as pathogenicity, stress responses and morphogenesis of fungal fruiting body. Fungal laccases have been noted to possess a broad substrate range, and thus have found applications in detoxification, wastewater treatment, and decolorization of industrial effluents. According to Shleev et al. (2004), the high redox potential (E°) of fungal laccases is one of the major factors responsible for their remarkable capability to oxidize substrates with high *E*° (*E*° > 400 mV), making them biocatalysts of special interest in the bioremediation of polycyclic aromatics, phenolic compounds as well as plastics. Thus, different studies have highlighted the biodegradation of PhACs by fungal laccases with the most remarkable results being recorded with laccases from the genus *Trametes*. For example, *T. versicolor* laccases was shown to bioremediate the antibiotics tetracycline, chlortetracycline, doxycycline and oxytetracycline (Suda et al., 2012). Similarly, the enzyme sourced from another specie of the same genus, *Trametes polyzona* was also recorded to degrade tetracycline, and some β-lactam, and quinolone antibiotics under redox mediator-free system (Lueangjaroenkit et al., 2019). Fungal laccases working synergistically were also shown to degrade PhACs. For example, the enzyme sourced from *T. versicolor, Myceliophthora thermophila* successfully biodegraded steroid hormones including estrone, 17ß-estradiol, estriol and 17α-ethinyl estradiol even at low enzyme activity (Becker et al., 2017).

### 4.2. Peroxidases

Peroxidases (EC 1.11.1.X) are majorly classified into the ascorbate-, cytochrome *c* catalase-, lignin-, manganese- and versatile peroxidases (Basumatary et al., 2023). Peroxidases sourced from fungi are noted to possess wide substrate specificity which enables them to catalyse the transformation of various recalcitrant compounds that are resistant to conventional bioremediation which includes synthetic dyes, herbicides, pesticides, and PhACs, to mention a few (Saikia et al., 2022). The various classes of peroxidases have been shown in different studies to biodegrade different PhACs. For example, *T. polyzona* manganese peroxidase was highlighted for its ability to degrade tetracycline, β-lactam, and quinolone classes with preferences for oxidizing dimethoxyl substituted phenol at the ortho-position (Lueangjaroenkit et al., 2019). A versatile peroxidase from *Bjerkandera adusta* was earlier recorded to degrade 100% diclofenac and oestrogens at very low enzyme concentration, in addition, the peroxidase also bioremediated 80% sulfamethoxazole and naproxen (Eibes et al., 2011). The steroid hormones such as the synthetic estrogen 17 α-ethinylestradiol, were significantly degraded by manganese peroxidase from *Pleurotus* spp. (Santosa et al., 2012). Similarly, in the study by Wen et al. (2009), lignin peroxidase sourced from *P. chrysosporium* was utilized in the *in vitro* degradation of tetracycline and oxytetracycline, achieving almost 100% removal rate within 5 min.

### 4.3. Monooxygenases

Fungal monooxygenases are important members of the superfamily of fungal oxidoreductases which have been demonstrated with the ability to catalyse biological oxidation/reduction reactions of various substrates. The fungal monooxygenases are quite ubiquitous in nature, and they generally facilitate different types of oxygen insertion reactions requiring two reductants, hence the nomenclature mixed function oxidases (Hussain et al., 2020). They have since been noted to be highly versatile with diverse applications in biotechnology, medicine, food and bioremediation including biodegradation of PhACs (Durairaj et al., 2016). Being the most effective fungal source of bioremediating enzymes, *T. versicolor* was shown to secrete a monooxygenase which remarkably degraded norfloxacin and ciprofloxacin (Prieto et al., 2011). Monooxygenases from other fungal species have also showed significant potential in the removal of PhACs. For instance, the biodegradation of the highly recalcitrant drug, carbamazepine to the less toxic 10,11-epoxycarbamazepine by the monooxygenases sourced from *P. ostreatus* was previously described by Golan-Rozen et al. (2011). The study further showed that the bioremediating activity of the enzyme on carbamazepine was significantly enhanced by the activity of an accessory enzyme, manganese peroxidase, from the same fungus (Golan-Rozen et al., 2011). More recently, a cytochrome P450 monooxyenase from *Phanerochaete chrysosporium*, another white-rot fungus catalysed the biodegradation of acetamiprid via N-adelkylation reaction mechanism (Mori et al., 2021). Fungal monooxygenases from various species have also been described to act in consortium for increased efficiency of PhACs removal. The monooxygenases from *P. chrysosporium* and *Pycnoporus sanguineus* which acted in synergy to remove ciprofloxacin, norfloxacin and sulfamethoxazole at a removal rate of 98.5 96.4 and 100%, respectively (Gao et al., 2018).

## 5. Mechanism of fungal bioremediation of PhACs

The amount of PhACs that eventually ends up in the environment varies according to many factors including the route of administration, the mode of metabolism within the patient as well as the route of excretion. For example, the excreted amount of antibiotics such as β-lactams, fluoroquinolones and tetracyclines was noted to be more than half of the administered dose (Berkner et al., 2014). On the other hand, even though lower fraction of macrolides, such as erythromycin, clarithromycin, azithromycin, fidaxomicin were excreted, these classes of pharmaceuticals are relatively more stable and thus persist longer in the environment (Booth et al., 2020). While in the environment, these PhACs partition into various compartments dependent on their physio-chemical properties and may undergo further transformation by abiotic or biological processes (Berkner et al., 2014). This has informed the exploration of fungi, amongst many other approaches, as natural, eco-friendly, sustainable, and cost-effective alternatives for PhACs biodegradation (Tomasini and León-Santiesteban, 2019).

Generally, the mechanisms behind the fungal bioremediation of PhACs in the environment can be summarized into four stages (Akerman-Sanchez and Rojas-Jimenez, 2021). These mechanisms/stages may be deployed singly, in synergy or sequentially to achieve PhACs degradation. At the first stage, the fungi, with the aid of their hyphae absorbs PhACs from the environment and immobilizes them in the cell (Akerman-Sanchez and Rojas-Jimenez, 2021). The fungal hyphae provide the mechanical strength for substrate penetration, increases contact surface area and assimilation of compounds from the environment, and co-generation of necessary enzyme systems (Gómez-Toribio et al., 2009). The second mechanism involves reactive oxygen species production by the fungal cells, which include, superoxide, hydroxyl reactive radical species, and peroxides (Akerman-Sanchez and Rojas-Jimenez, 2021). The well-known Fenton reactions in fungi has been speculated to be linked to PhACs biodegradation as the high, non-specific redox potential of generated radicals in the pathway render them efficacious in breakdown of a range of PhACs (Gómez-Toribio et al., 2009). For instance, radicals from the Fenton reaction in *Pleurotus eryngii* were shown to enhance dismutation and laccase oxidation of aromatic aldehyde and hydroquinone (Gómez-Toribio et al., 2009).

In the third mechanism, an array of extracellular fungal enzymes is expressed, this could include phenol-oxidases (laccases) and peroxidases (versatile, lignin, manganese peroxidases), among others (Rodríguez-Rodríguez et al., 2013). Interestingly, a recent study demonstrated the ability a dye decolorizing peroxidase 4 (DyP4), to efficiently breakdown PhACs like furosemide and paracetamol, with enhanced degradation of sulfamethoxazole, salicylic acid and methyl paraben in the presence of redox mediators (Athamneh et al., 2022). Although DyPs, a group of heme-containing peroxidases derivable from fungi, bacteria and archaea, they were first known for dye breakdown, however, their hydrolytic and oxidative capabilities are also believed to be closely linked to lignin degradation (Athamneh et al., 2022). The fourth mechanism involves the deployment of intracellular enzymes, most especially, the cytochrome P450 (CyP) complex (Asif et al., 2017). The CyP complex coupled with extracellular enzymes mediate the processes of hydroxylation, dehalogenation, deamination and dealkinalization, changing the structure of pollutants and enhancing mineralization (Asif et al., 2017). The CyP epoxide hydrolases, transferases and monooxygenases complex are of special relevance in PhACs bioconversion. Overall, both intra- and extra-cellular enzymes, hyphal mass branching and ROS generation are believed in many instances to work together for PhACs decontamination (Chandra and Chowdhary, 2015; Naghdi et al., 2018). Studies have shown the fungi *Cunninghamella echinulate* taking up the analgesic, paracetamol, and biotransforming it into N-acetyl-p-benzoquinoneimine through rearrangement and hydroxylation pathways (Kumari et al., 2009). Similarly, *Aspergillus niger* has been demonstrated to be capable of metabolizing naproxen into less harmful by-products via the hydroxylation mechanism (He and Rosazza, 2003). The utilization of decarboxylation (Parshikov et al., 2002) and metal ion chelation reactions have also been reported for certain ascomycete fungi in the genus *Trichoderma* and *Fusarium* (Tigini et al., 2014).

The production of fungal bio-surfactants has also been shown to play a role in the bioremediation of PhACs (Cicatiello et al., 2016). As amphiphilic surface-active compounds, fungal biosurfactants improve molecular interactions by decreasing the interfacial tension; these surfactants which are made up of various types of lipids, polysaccharides and protein-lipids complexes enhance bioremediation processes by increasing the bioavailability and mobility of PhACs (Olicón-Hernández et al., 2017). As mechanistic pathways in mycoremediation of PhACs continue to evolve, it has been suggested that the process may need to be merged with nanotechnology (coupling basidiomycete fungi or their enzymes with nano-materials in varied bioreactor designs), protein engineering strategies, physicochemical and ‘omic’ techniques (Meganathan et al., 2021).

## 6. Fungal bioreactors for PhACs degradation

Bioreactors are enclosed vessels where biological reactions take place and they have been identified as one of the most remarkable technologies for the treatment of a variety of wastewaters (Mishra et al., 2022). Within these vessels, microorganisms such as bacteria, fungi, or algae, consume and degrade the toxic pollutants in the effluent, converting them into less harmful derivatives and sometimes basic compounds such as carbon dioxide and water (Khalidi-idrissi et al., 2023). Bioreactors have been noted to have various advantages over conventional methods of removing PhACs as they create less sludge, have lower energy demand and typically emit lower amounts of greenhouse gases (Dos Santos et al., 2022). As a result of this, bioreactor, including those with fungi components, have become part of contemporary pharmaceutical wastewater treatment system and their potential to bioremediate PhACs have been the focus of many studies (Cruz-Morató et al., 2014; Tormo-Budowski et al., 2021; Dos Santos et al., 2022). In this regard, different types of bioreactor configurations that have been noted for their efficiency in the treatment of PhACs using either the whole fungus or the enzyme (s); these include membrane bioreactors, sequencing batch reactors, fluidized bioreactors, etc. (Mishra et al., 2022). Although whole-cell fungal cultures have been employed for the removal of PhACs in both submerged and solid bioreactor conditions, it was observed that submerged whole-cell cultures have been more reported (Tiwari et al., 2021). Typically, the operation of fungal bioreactors take place in continuous, semi-batch, batch systems, and under anaerobic or aerobic conditions. Systems involving submerged growth include the airlift, bubble column, packed bed, and stirred tank (most used with mechanical agitation, good aeration, and fluid mixture). Also, aerated suspended air-lift loop or fluidized-bed reactors are useful for growing fungal species that from pellets, enhancing fungi recoverability (Crognale et al., 2002). However, agitated reactors may also produce high energy requirements from increased agitation speed, and stressed environments with adverse effects on microbial growth. Unlike the stirred tank system, in cylindrical air-lift reactors where air, gas or oxygen is injected from the reactor base, less microbial stress is generated (Zhong, 2011).

In specific examples, the degradation of carbamazepine by *T. versicolor* took place in an air-pulsed fluidized myco-reactor, aqueous media and in both continuous and batch culture models (Jelic et al., 2012). Likewise, *P. chrysosporium* also broke down carbamazepine in a continuous and batch plate bioreactor system, however, it was highly nutrient-dependent (Zhang and Geißen, 2012). Trickle filter and hollow fibre membrane bioreactor technologies based on immobilized *T. versicolor* were noted to be applicable as large scale, inexpensive options for continuous wastewater treatment. Generally*, T. versicolor* was observed to be a model fungus, serving as the biological component in many of these fungal bioreactors. This assertion is due to the fact that the fungal specie along with its various enzymes, is the most recurrent biological components utilized for the effective bioremediation of emerging contaminants, which is ascribed to their highly advanced enzymatic machinery (Cruz-Morató et al., 2014). For example, *T. versicolor* was utilized in a fluidized bed bioreactor which effectively removed ~80% of the PhACs, including ciprofloxacin, clarithromycin, codeine, diclofenac, naproxen, ibuprofen, ketoprofen, ofloxacin, phenazone, and salicylic acid, in the hospital effluent within 8 days (Cruz-Morató et al., 2014). Earlier, this model fungus was shown in another fluidized bed bioreactor to degrade 70% of PhACs, mainly carbamazepine in the wastewater (Cruz-Morató et al., 2013). *T. versicolor* immobilized in a stirred tank bioreactor was also able to significantly eliminate 16 PhACs in synthetic wastewater and those naturally present in the hospital wastewater at a removal rate of 95.7 and 85.0%, respectively (Tormo-Budowski et al., 2021). However, many other fungi, besides *T. versicolor* have been successfully utilized in different bioreactor configurations for PhACs bioremediation. A notable example was demonstrated in the study by Zhang and Geißen (2012), where *P. chrysosporium*, another white rot fungus was employed in a plate bioreactor and operated in both sequence batch and continuous modes to eliminate carbamazepine at significant removal rates of between 60 and 80%. The study further noted that the bioreactor was effective while working continuously for close to 100 days. Apart from white-rot fungus, *Penicillium oxalicum*, an ascomycete, was used for the bioremediation of diclofenac in a bench bioreactor, with the activation of the cytochrome P450 enzymes playing prominent roles in its bioremediation effort (Olicón-Hernández et al., 2019).

Interestingly, fungal cultures working in consortium have also been demonstrated to achieve higher efficiency due to their synergistic bioremediative effects. In this regard, a fungal consortium containing *Aspergillus niger, Mucor circinelloides, Trichoderma longibrachiatum, Trametes polyzona* and *Rhizopus microspores* was applied in a stirred fluidized bioreactor for the simultaneous biodegradation of carbamazepine, diclofenac, and ibuprofen as well as their transformation metabolites (Kasonga et al., 2020). Similarly, a trickle-bed bioreactor based on various fungal biomass immobilized on rice husks was recently shown to achieve an elimination of 88.6 and 89.8% in synthetic and real wastewater, respectively, and it was also shown that adsorption was an important physical phenomenon in the PhACs elimination effectiveness of the trickle-bed bioreactor (Tormo-Budowski et al., 2021). However, the degradation of PhACs by bioreactor based on fungal organism has been noted to be significantly enhanced by external supplementation of nutrients and increased aeration (Badia-Fabregat et al., 2015).

In addition, successful attempts have been made at using enzymes rather than whole fungal organism as the biological component of the bioreactor (Khalidi-idrissi et al., 2023). For instance, cross-linked enzymes aggregates of laccase and polysulfone hollow fiber membrane was developed for the elimination of acetaminophen, carbamazepine and mefenamic acid (Ba et al., 2014). However, a major challenge in fungal-based bioreactors for PhACs removal is bacterial contamination which reduces PhACs removal effectiveness as bacteria compete with fungi for growth substrates, disturb fungi growth, and destroy the mycelium (Shi et al., 2019). As a result, developing strategies for continuous fungal growth is critical. Some effective measures for avoiding bacterial contamination have been identified to include the lowering the pH of the reaction to an acidic range, immobilizing the fungus, limiting the nitrogen concentration in growth medium, the use of mild disinfectants, as well as pretreatment of wastewater, especially sterilization (Shi et al., 2019). In other instances, however, the operation of bioreactors under unsterilized conditions has also been shown to be effective in the removal of PhACs, this was ascribed to the synergy between the bioremediative abilities of the fungi and natural bacteria population present in the wastewater (Yang et al., 2013; Kasonga et al., 2020). For example, in a membrane bioreactor based on *T. versicolor*, operation under unsterilized conditions showed a removal rate of approximately 55 and 90% for diclofenac and bisphenol A at a loading rate of ~500 μg/L.d (Yang et al., 2013). This was also shown in a previous study in which a strain of *Penicillium oxalicum* removed significant amount of acetaminophen, acetylsalicylic acid, diclofenac, ibuprofen, ketoprofen, mefenamic acid, naproxen, trimethoprim with the aid of the natural bacterial consortium in the hospital wastewater (Olicón-Hernández et al., 2021).

## 7. Conclusion and future directions

In this paper, the potential of fungi as promising tools for PhACs bioremediation, which is due to their versatile metabolic capabilities, has been established, however, there are still a lot of gaps to be filled in order to realize the full potential of this biotechnological approach. There remains the need for further research on enhancing the fungal bioremediative activity and scaling up of the process for industrial applications. It is believed that currently less than 10% of the total fungi on earth have been curated for their importance as tools in biotechnological field, thus signifying a gold mine for further exploration. In this regard, the identification of new fungi species with a high capability of degrading pharmaceutical compounds would go a long way in enhancing the efficiency of PhACs bioremediation. Similarly, the screening, identification, and characterization of novel enzymes for PhACs biodegradation is another possible avenue of advancing this field of biotechnology. Unraveling the metabolism of the various fungi is also key in understanding the downstream pathways for fungal bioremediation of PhACs and the mechanisms involved in the reactions. Gaining insights into key biodegradative pathways of environmentally important fungi has become more feasible with the advent of in Next-Generation sequencing as well as various databases like MetaCyc and the KEGG pathway database. Similarly, techniques such as metagenomics, meta-transcriptomics, meta-proteomics, and other omic methods can aid in gaining a better understanding of the interactions within microbial communities by elucidating the genomic organization of these communities and identifying the various genes that participate in bioremediation. As have been well demonstrated in other areas of biotechnology, the development of genetic tools for fungi can also enhance the engineering of fungi strains with enhanced bioremediation capabilities of PhACs. These tools can be deployed in the production of recombinant enzymes, manipulation of pathways, directed evolution of proteins as well as the mutation of fungi and their proteins amongst many other approaches. Gene editing is a fast-growing approach being currently employed to manipulate DNA with the aim of creating or modifying organisms that are better suited for definite bioprocesses, however, this has not been fully explored in fungal bioremediation. Currently, CRISPR-Cas (Clustered Regularly Interspaced Short Palindromic Repeats-CRISPR associated protein 9), ZFN (Zinc Finger Nuclease) and TALEN (Transcription Activator-Like Effector Nucleases) are the major gene editing tools, which if fully deployed, possess the capacity to improve the bioremediation processes in fungi. Optimization of key process parameters in any bioprocess has been noted to be critical in increasing efficiency and it can be achieved via the one factor at a time approach, and through statistical and mathematical approaches such as response surface methodology, neural networks, etc. While it is expedient to explore all of the possibilities highlighted above, it is even more important that the ecological impact of fungal bioremediation on the environment must be fully assessed to ensure the sustainability of this approach. Overall, the use of fungi and their enzymes for PACs degradation has great potentials to mitigate the environmental impact of pharmaceutical waste while contributing to sustainable waste management practices.

## Author contributions

AA contributed to conception, original writing, and editing of the manuscript. CA, SA, JO, and TDA contributed to original writing and editing of the manuscript. JL contributed to conception and editing of the manuscript as well as fund acquisitions. All authors contributed to the article and approved the submitted version.

## Funding

This work was supported by a Universiti Sains Malaysia, Special Short-Term Grant with Project No: 304/PTEKIND/6315670.

## Conflict of interest

The authors declare that the research was conducted in the absence of any commercial or financial relationships that could be construed as a potential conflict of interest.

## Publisher’s note

All claims expressed in this article are solely those of the authors and do not necessarily represent those of their affiliated organizations, or those of the publisher, the editors and the reviewers. Any product that may be evaluated in this article, or claim that may be made by its manufacturer, is not guaranteed or endorsed by the publisher.
